# A Closed-Loop Audit of Early Mobilization of Thoracic Surgery Patients on Postoperative Day 0

**DOI:** 10.7759/cureus.73020

**Published:** 2024-11-04

**Authors:** Elif Demirtas, Thomas Brookes

**Affiliations:** 1 Surgery, University Hospitals Birmingham, Birmingham, GBR; 2 Thoracic Surgery, University Hospitals Birmingham, Birmingham, GBR

**Keywords:** clinical audit, early mobilization, enhanced recovery pathways (eras), postoperative day zero, thoracic surgery

## Abstract

Introduction

Early mobilization in the postoperative period, particularly on the day of surgery, is crucial for reducing morbidity and mortality in thoracic surgery patients. Starting physical activity as soon as clinically feasible enhances recovery and is a key component of enhanced recovery pathways. Effective implementation of early mobilization protocols requires collaboration among multidisciplinary teams (MDT), emphasizing its importance in postoperative care. This audit evaluates adherence to early mobility protocols for thoracic surgery patients at Queen Elizabeth Hospital, Birmingham (QEHB), focusing on mobilization rates on postoperative day 0 (POD-0) in line with the Enhanced Recovery After Surgery* *(ERAS) guidelines. It also aims to identify barriers to optimal practice and improve current practice through targeted interventions, including MDT training and posters to enhance healthcare professionals' adherence.

Methods

This closed-loop audit at QEHB targeted thoracic surgery patients from November 2023 to June 2024. Data collection occurred in three cycles: the first from November to December 2023 (60 patients), the second from February to March 2024 (59 patients), and the third from May to June 2024 (39 patients). Retrospective data were collected using an electronic documentation system, capturing demographic and clinical variables, including age, sex, preoperative mobility, comorbidities, and surgical details. The primary outcomes assessed were POD-0 mobilization rates and mobility levels achieved. Interventions included MDT training on ERAS guidelines and reminder posters for doctors. Data were analyzed by using descriptive statistics for demographic and clinical variables. Additionally, regression analysis was performed to assess the impact of interventions on mobilization rates on POD-0.

Results

Pre-intervention data showed that only 30% (n=18) of patients mobilized on POD-0. Ward patients had a 44% (n=15) mobilization rate, while rates were lower in the high dependency unit (HDU) and enhanced post-operative care (EPOC) at 16.7% (n=3) and 0% (n=0), respectively. After two interventions (MDT training and posters), mobilization on POD-0 increased to 45.8% (n=27) and 67.6% (n=25) in subsequent cycles. Among ward patients mobilization on POD-0 increased to 94.1% (n=16), while HDU patients improved to 55.5% (n=5). Additionally, it varied by surgical approach, with robotic-assisted thoracoscopic surgery (RATS) patients rising from 30% (n=6) to 45.5% (n=15) and video-assisted thoracoscopic surgery (VATS) patients increasing from 23.8% (n=5) to 81.8% (n=9) at the end of the third cycle. Patients mobilizing on POD-0 had a shorter median length of stay (two days; interquartile range (IQR)=3) compared to those who did not (five days; IQR=5). Confounding factors were not considered, so no causal conclusions should be drawn.

Conclusion

This audit demonstrates the successful implementation of early mobilization protocols for thoracic surgery patients at QEHB, highlighting the positive impact of targeted interventions on adherence to ERAS guidelines. Initially, only 30% of patients mobilized on POD-0, but this improved to 67.6% after interventions. This notable increase highlights the importance of coordinated efforts among healthcare professionals in promoting early mobilization to enhance patient outcomes post-surgery.

## Introduction

Early postoperative mobilization has emerged as a key practice in surgical procedures across multiple surgical specialities, including general surgery, trauma and orthopaedics, and thoracic surgery, and aims to reduce postoperative morbidity and mortality. This practice is linked to both the physiological and psychological benefits of early mobilization, making it an essential component of modern recovery protocols [1-4]. Early mobilization is defined as the initiation of movement and physical activity shortly after surgery, often within the first 24 hours [5,6]. It can involve actions such as sitting on a chair, standing up from a chair, doing exercises in or out of bed, and walking within the room or hallway [1]. The physiological benefits of early mobilization are primarily related to preventing complications that arise from immobility, such as pulmonary issues (atelectasis and pneumonia), venous thromboembolism, and muscle atrophy [7-9]. Current evidence shows the effectiveness of early mobilization in various medical settings, including intensive care units, where it plays a critical role in reducing muscle weakness caused by prolonged immobility [9,10], as well as in postoperative management for various surgeries, such as laparotomies in general surgery or total hip replacements in orthopaedics [1,2]. Considering this, early mobilization is particularly crucial for thoracic surgery patients, as lack of mobility can lead to respiratory complications and other related issues. For instance, a study by Rogers et al. on patients undergoing lung cancer resections demonstrated that early mobilization significantly reduces morbidity and hospital length of stay [4]. Additionally, the Enhanced Recovery After Surgery (ERAS) guidelines for thoracic surgery emphasize the importance of mobilizing patients as early as possible after surgery to facilitate faster recovery and minimize the risk of complications [3]. Although early mobilization is strongly recommended and generally considered safe, the quality of the evidence supporting its effectiveness remains somewhat limited [2,3]. This highlights the need for higher-quality studies to further investigate this practice and strengthen the available evidence.

The successful implementation of early mobilization protocols heavily relies on a multidisciplinary approach requiring close coordination among healthcare providers, including physical therapists, nurses, and surgeons, as well as active engagement of patients [11,12]. While few studies have explored the factors influencing early mobilization adoption, certain barriers, both institutional (such as nurse-to-patient ratios) and patient-specific (including surgical complications and preoperative conditions), hinder consistent implementation [11,12]. A qualitative study done by Ni et al. highlighted the crucial role healthcare professionals play in encouraging patient participation, demonstrating that perioperative support can significantly enhance mobilization efforts [12]. Given that, when aligned with ERAS protocols, early mobilization, requiring a coordinated multidisciplinary approach to ensure its timely and efficient implementation, can serve as a vital intervention to enhance recovery and improve patient outcomes in thoracic surgery.

Building on the above, this audit aims to evaluate the implementation of early mobility protocols for thoracic surgery patients at Queen Elizabeth Hospital, Birmingham (QEHB). The primary objective is to assess adherence to the early mobilization recommendations outlined in the ERAS guidelines and to identify specific factors that may hinder optimal practice on postoperative day 0 (POD-0). Additionally, the audit seeks to improve current practice in thoracic surgery wards at QEHB with ERAS early mobilization standards through targeted interventions, including multidisciplinary team (MDT) training and the use of posters, to improve adherence among healthcare professionals to facilitate effective implementation.

The project was presented as a poster at the West Midlands Surgical Society Conference on November 1, 2024.

## Materials and methods

A retrospective three-cycle audit was conducted from November 2023 to June 2024 as part of clinical governance within the QEHB thoracic surgery department. Registered with the University Hospitals Birmingham Clinical Governance and Patient Safety Team, this audit used anonymized data from routine clinical care, thus waiving the need for patient consent or ethical approval.

The audit included all patients who underwent elective or emergency thoracic surgery at QEHB. Patients managed conservatively without surgery (e.g., for rib fractures, pneumothorax) were excluded. Data collection was performed retrospectively using the electronic documentation system by the authors.

Data were collected on both demographic and clinical variables; sex, age, preoperative mobility (included preoperative Eastern Cooperative Oncology Group (ECOG) performance status), and comorbidities affecting mobilization (e.g., cardiac and pulmonary conditions, neurological conditions, osteoarthritis). Additional variables included the surgical approach (robotic-assisted thoracoscopic surgery (RATS), video-assisted thoracoscopic surgery (VATS), and thoracotomy) and the type of surgery performed. The primary outcomes measured were whether the patient mobilized on POD-0 and the level of mobility achieved, categorized in the second and third cycles as follows: sitting out on a chair (M1), mobilizing to the bathroom (M2), and mobilizing in the corridor or beyond (M3). Other variables included the presence of a chest drain, the type of analgesia used (epidural, paravertebral block (PVB), patient-controlled analgesia (PCA), or oral analgesia), and the postoperative location (such as ward bed, high-dependency unit (HDU) bed, Enhanced Postoperative Care (EPOC) Unit, or Intensive Therapy Unit (ITU)). Additionally, we recorded the timing of arrival from the recovery room, whether early mobilization was emphasized in postoperative assessment notes by doctors, and whether mobilization was led by nurses or physiotherapists. Other factors included length of stay, chest drain removal (if applicable), admission to the ITU, complications, and readmissions after discharge.

Two key interventions were implemented during the audit. Following the first cycle, MDT teaching on ERAS guidelines was introduced to encourage early mobilization. After the second cycle, posters were implemented to remind resident doctors to promote early mobilization during postoperative assessments.

Following data collection, surgery types were categorized based on procedural similarities as follows: lobectomy/segmentectomy, wedge resections/bullectomy/lung volume reduction surgery (LVRS), washout/decortication, bronchoscopy/cervical mediastinoscopy/navigational bronchoscopy, anterior mass resections, and other. Preoperative comorbidities were classified as no comorbidity, one comorbidity, and two or more comorbidities. Data were analyzed using Statistical Product and Service Solutions (SPSS, version 29.0; IBM SPSS Statistics for Windows, Armonk, NY). Descriptive statistics were used to summarize patient demographics and clinical details. Counts and percentages were calculated for categorical variables, such as sex, comorbidities, and the mobilized patients on POD-0, mobilization levels while continuous variables, such as age and length of stay, are expressed as median and interquartile range (IQR). The distribution of data was assessed with the Shapiro-Wilk test. A p-value below 0.05 is considered statistically significant. A multiple logistic regression analysis was conducted to evaluate the impact of various interventions on mobilization rates on POD-0, following the identification of other potential factors influencing mobilization on POD-0 through univariate analysis. The regression model included the following covariates: audit cycles, surgical approach, type of surgery, and type of analgesia administered. The Hosmer-Lemeshow test was employed to assess the model's goodness of fit. Although descriptive statistics were reported for the length of stay, no statistical comparisons or regression analyses were performed due to the inability to account for confounding factors. In this study, pairwise deletion was used to manage missing data, allowing for the inclusion of all available information in each analysis while preserving the integrity of the results.

This audit provides valuable insights into early mobilization practices within thoracic surgery at QEHB and highlights the impact of MDT interventions on patient outcomes.

## Results

A total of 156 patients were included in the audit, with 60 patients in the first cycle, 59 in the second, and 37 in the third. The median age of patients was 65, 64, and 68 years in each respective cycle, each with an IQR of 19. Female patients accounted for 46% (n=28) of the first cycle, 49.2% (n=29) of the second cycle, and 56.2% (n=21) of the third cycle. Preoperative mobility and ECOG performance scores showed that 93.3% (n=56) of patients in the first cycle were independently mobile, compared to 84.9% (n=45) in the second cycle and 100% (n=37) in the third. Additionally, 46.6% (n=28) of patients in the first cycle had two or more comorbidities, increasing to 54.3% (n=32) in the second and 75.6% (n=28) in the third cycle.

RATS was performed in 33.3% (n=20) in the first cycle, 42.4% (n=25) in the second, and 29.7% (n=11) in the third. VATS was conducted in 35% (n=21), 30.5% (n=18), and 29.7% (n=11) of cases, respectively, across the same cycles. Lobectomy and segmentectomy procedures were performed in approximately 40% of cases across all cycles. In the third cycle, the proportion of wedge resections was lower compared to the previous cycles, while bronchoscopy and cervical mediastinoscopy accounted for 24.3% (n=9) of procedures. Further details, including operation side, and analgesia type are provided in Table 1.

**Table 1:** Clinical and demographic characteristics >POD-0: Patients who did not mobilize on POD-0, Mobilized POD-0: Patients who mobilized on POD-0, ECOG: Eastern Cooperative Oncology Group RATS: Robotic-Assisted Thoracoscopic Surgery, VATS: Video-Assisted Thoracoscopic Surgery, Other: Includes bronchoscopic approaches and incision types not categorized under the main groups, LVRS: Lung Volume Reduction Surgery, PCA: Patient-Controlled Analgesia, PVB: Paravertebral Block

Clinical and Demographic Characteristics		1st Cycle		2nd Cycle		3rd Cycle	
		n	%	n	%	n	%
Sex	Female	28	46.7	29	49.2	21	56.8
	Male	32	53.3	30	50.8	16	43.2
POD-0 Mobility	>POD-0	42	70	32	54.2	12	32.4
	Mobilised POD-0	18	30	27	45.8	25	67.6
Performance Status	ECOG 0	41	68.3	37	69.8	18	48.6
	ECOG 1	14	23.3	13	24.5	16	43.2
	ECOG 3	5	8.3	3	5.7	3	8.1
Pre-op Mobility	Fully Mobile	56	93.3	45	84.9	37	100
	Walks with stick/frame	4	6.7	8	15.1	0	0
	Unable to walk	0	0	0	0	0	0
Comorbidities	No comorbidities	16	26.7	13	22	4	10.8
	1 comorbidity	16	26.7	14	23.7	5	13.5
	2 or more comorbidities	28	46.6	32	54.3	28	75.6
Incision	RATS	20	33.3	25	42.4	11	29.7
	VATS	21	35	18	30.5	11	29.7
	Thoracotomy	7	11.7	6	10.2	5	13.5
	Other	12	20	10	16.9	10	27
Surgery	Lobectomy/Segmentectomy	24	40	23	39	15	40.5
	Wedge Resections/Bullectomy/LVRS	16	26.7	17	28.8	8	21.6
	Washout/Decortication	2	3.3	3	5.1	3	8.1
	Bronchoscopy/Cervical Mediastinoscopy/Navigational Bronchoscopy	9	15	10	17	9	24.3
	Anterior mass resection	5	8.3	3	5.1	1	2.7
	Others	4	6.7	3	5.1	1	2.7
Side	Left	25	41.7	23	39	8	21.6
	Right	28	46.7	26	44.1	19	51.4
Analgesia Type	Oral Analgesia	23	38.3	10	16.9	13	36.1
	PCA	10	16.7	9	15.3	4	11.1
	PVB	25	41.7	35	59.3	17	47.2
	Epidural	2	3.3	5	8.5	2	5.6

Early mobilization on POD-0

**Figure 1:**
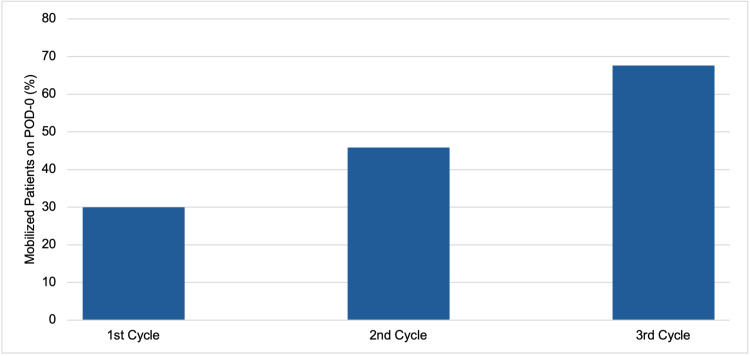
Early mobilization rates across cycles

Pre-intervention data showed that only 30% (n=18) of patients mobilized on POD-0, which was well below the ERAS guideline standards. After the introduction of MDT teaching and poster campaigns, the mobilization rate significantly improved, rising to 45.8% (n=27) in the second cycle and further to 67.6% (n=25) in the third, as shown in Figure 1. Regression analysis indicated a strong association between the implementation of these interventions and improved POD-0 mobilization rates (OR=10.92, 95% confidence interval (CI): 3.27-36.42, p<0.05) (Table 2). Additional findings on other potential influencing variables, including surgery type, surgical approach, and analgesia type (which demonstrated statistical significance in univariate analysis and were thus included in the regression analysis), are available in Table 3. However, since this is an observational study, these associations should not be interpreted as causal and may be limited by potential confounding factors that were not included in this analysis.

**Table 2:** Regression analysis of audit cycles for early mobilization on POD-0 P-values below 0.05 are considered statistically significant. "—" indicates that the odds ratio (OR) and 95% confidence intervals (CI) are not applicable or could not be calculated for the respective category.

Covariates	p-value	Odds Ratio (OR)	95% Confidence Intervals (CI) for OR	
			Lower	Upper
Audit Cycles	0.001	-	-	-
2nd Cycle	0.061	2.67	0.955	7.467
3rd Cycle	0.000	10.92	3.273	36.425

**Table 3:** Regression analysis of other variables on early mobilization on POD-0 * Reference group accepted bronchoscopy/cervical mediastinoscopy/navigational bronchoscopy. P-values below 0.05 are considered statistically significant. "—" indicates that the odds ratio (OR) and 95% confidence intervals (CI) are not applicable or could not be calculated for the respective category.

Covariates	p-value	Odds Ratio (OR)	95% Confidence Intervals (CI) for OR	
			Lower	Upper
Surgery Type*	0.044	-	-	-
Lobectomy/Segmentectomy	0.004	0.002	0	0.149
Wedge Resections/Bullectomy/LVRS	0.019	0.008	0	0.45
Washout/Decortication	0.011	0.003	0	0.268
Anterior Mass Resections	0.017	0.005	0	0.386
Others	0.067	0.056	0.003	1.221
Surgical Approach	0.215	-	-	-
Analgesia Type	0.593	-	-	-

**Figure 2:**
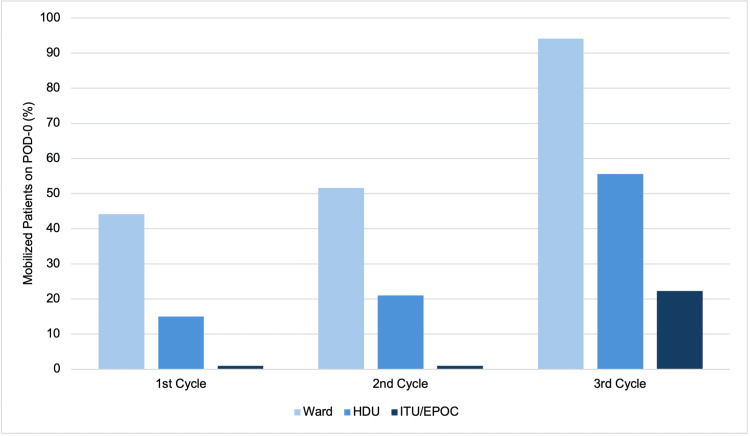
Early mobilization on POD-0 rates across postoperative locations

Early mobilization rates varied based on patient location. In each cycle patients in the ward consistently exhibited higher mobilization rates than those in HDU and ITU/EPOC. In the first cycle, 44.1% (n=15) of ward patients mobilized on POD-0, compared to 16.7% (n=3) in the HDU and 0% in the EPOC unit. In the second cycle, ward mobilization rose to 59.3% (n=16), while 21% (n=4) of patients in the HDU mobilized. By the third cycle, mobilization rates reached 94.1% (n=16) in the ward, 55.5% (n=5) among patients in the HDU, and 22.2% (n=2) in the ITU/EPOC (Figure 2).

**Figure 3:**
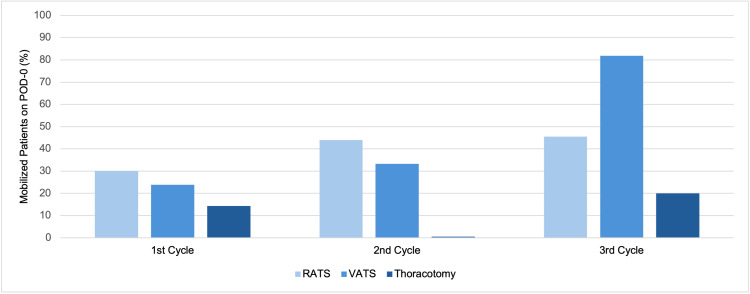
Early mobilization on POD-0 rates across surgical approaches RATS: Robotic-Assisted Thoracoscopic Surgery, VATS: Video-Assisted Thoracoscopic Surgery

In the first cycle, early mobilization was observed in 30% of RATS patients (n=6), compared to 23.8% (n=5) of VATS patients and 14.3% (n=1) of thoracotomy cases. In the second cycle, 44% (n=11) of RATS patients mobilized on POD-0, while 33.3% (n=6) of VATS patients did, and no thoracotomy patients achieved early mobilization. By the third cycle, mobilization rates improved significantly to 45.5% (n=5) of RATS patients, 81.8% (n=9) of VATS patients, and 20% (n=1) of thoracotomy patients mobilized on POD-0 (Figure 3). Specifically, for surgical types, there was a steady increase in mobilization rates across the three cycles for lobectomy/segmentectomy cases, from 8.7% (n=2) to 42.9% (n=6), and for wedge resections/bullectomy/LVRS cases, from 37.5% (n=6) to 75% (n=6) (Table 4).

**Table 4:** Early mobilization on POD-0 rates across surgical approach and surgery types RATS: Robotic-Assisted Thoracoscopic Surgery, VATS: Video-Assisted Thoracic Surgery, Other: Includes bronchoscopic approaches and incision types not categorized under the main groups, LVRS: Lung Volume Reduction Surgery

Mobilization on POD-0		1st Cycle		2nd Cycle		3rd Cycle	
		n	%	n	%	n	%
Surgical Approach	RATS	6	30.0	11	44.0	5	45.5
	VATS	5	23.8	6	33.3	9	81.8
	OPEN	1	14.3	0	0.0	1	20.0
	Other	6	50.0	10	100.0	8	100.0
Surgery Types	Lobectomy/Segmentectomy	2	8.7	6	26.1	6	42.9
	Wedge/Bullectomy/LVRS	6	37.5	9	52.9	6	75.0
	Washout/Decortication	1	50.0	0	0.0	2	66.7
	Bronchoscopy/Cervical Mediastinoscopy	6	66.7	10	100.0	8	100.0
	Anterior Mass	2	40.0	0	0.0	1	100.0
	Others	1	25.0	2	66.7	2	66.7

Among the patients who mobilized on POD-0 during the second cycle, 7% (n=2) achieved level M1 mobilization, 55% (n=16) reached level M2, and 31% (n=9) reached level M3. In the third cycle of the 25 patients who mobilized on POD-0, 20% (n=5) achieved level M1, 48% (n=12) reached level M2, and 32% (n=8) achieved level M3.

**Figure 4:**
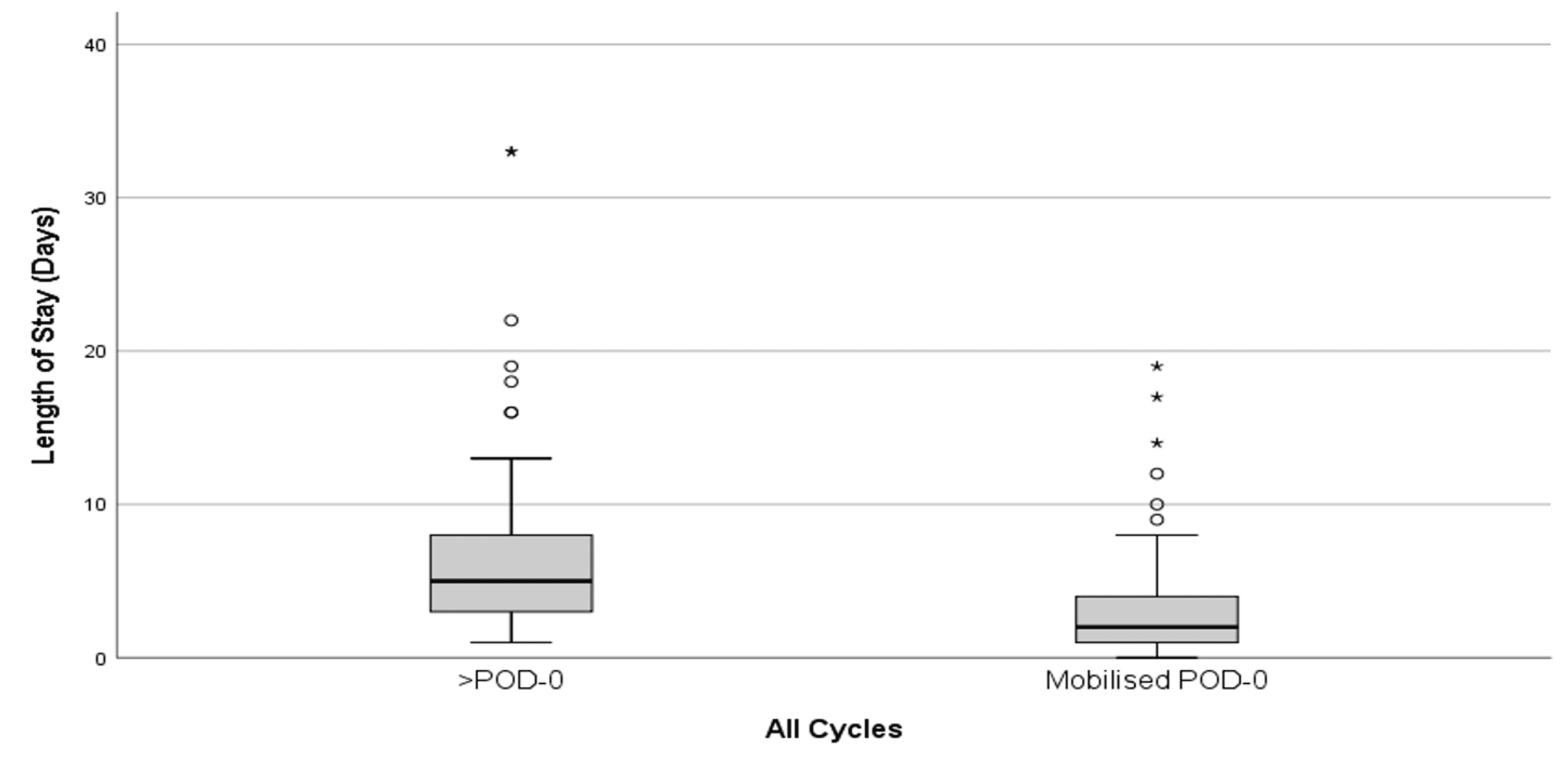
Length of stay comparison across early mobilization groups (all cycles) >POD-0: Patients who did not mobilize on POD-0; Mobilized POD-0: Patients who mobilized on POD-0 Note: 1. One patient (2nd cycle, >POD-0) had an extended stay of 77 days; this case is not shown in the graph. 2. One patient (3rd cycle, >POD-0) died while still an inpatient, and this is not shown in the graph.

For each cycle, patients who mobilized on POD-0 had a shorter median length of stay compared to those who did not. Overall, the median length of stay for patients who did not mobilize on POD-0 was five days (IQR=5), while, for those who mobilized on POD-0, it was two days (IQR=3) (Figure 4). Notably, one patient in the third cycle who was not mobilized on POD-0 died while still an inpatient, and another patient in the second cycle who was not mobilized on POD-0 had an extended stay of 77 days. It is important to note that confounding factors were not included in this analysis, and no causative implications should be drawn from these findings. Across all cycles, mobilization on POD-0 was significantly more likely among patients who returned from recovery before 17:00. All POD-0 mobilizations were facilitated by nursing staff.

## Discussion

The aim of this audit was to evaluate current practices at a large regional thoracic surgical tertiary center against national ERAS guidelines. Our findings revealed that adherence to early mobilization criteria in thoracic surgery patients at QEHB was initially poor, with only 30% (n=18) of patients meeting the standards in the first cycle. However, following targeted interventions, such as MDT teaching and poster reminders, compliance significantly improved over eight months, increasing to 67.6% (n=25). Our findings also showed that early mobilization rates differed according to various factors, including the time of patient arrival from recovery, their postoperative location, and patient- and surgery-specific characteristics. Our findings emphasize the crucial role of healthcare professionals in promoting early mobilization through multidisciplinary team education and awareness campaigns, which have been emphasized in the literature as key factors in improving postoperative care [12,13]. However, it is worth noting that this audit lacked patient-centered interventions, such as strategies aimed at directly increasing patient engagement in early mobilization, despite patient perspectives being recognized in the literature as a crucial factor for success [12]. This represents an area for further exploration in future audit projects.

This audit achieved broad outreach across different patient care areas (ward, HDU, and ITU/EPOC), showing that the interventions were able to engage and educate a wide range of healthcare professionals involved in patient care. On the other hand, the increase in early mobilization rates was more pronounced among VATS patients compared to RATS or thoracotomy. This suggests that our interventions may not have fully addressed patient-specific factors related to different surgical procedures, highlighting the need for further tailored interventions focused on both the type of surgery and individual patient needs within the thoracic surgery department. Another notable finding was that all patients who mobilized on POD-0 were assisted by nursing staff rather than physiotherapists. This finding shows the importance of further supporting nursing staff to enhance adherence to early mobilization protocols, which could lead to even greater improvements.

While we found an increased early mobilization following our interventions, it is important to note that this association was primarily observed descriptively and may have been influenced by the limitations of our audit. One of the limitations of our audit is that data collection relied on an electronic documentation system; if early mobilization was not documented for a patient, they were classified as not mobilized on POD-0. Additionally, after MDT training and poster implementation, there may have been increased awareness regarding the documentation of mobility status among healthcare professionals, which was likely absent in the first audit cycle. These could lead to a higher number of patients recorded as not mobilized on POD-0 in our audit compared to the actual situation. Additionally, we must acknowledge the limitations of our regression analysis; our ability to assess the true impact of other factors included in the analysis is limited by sample size, study design, and potential confounding variables that were not accounted for in our audit. Consequently, the findings from our regression analysis regarding other covariates should be interpreted with caution. Furthermore, since this is an observational study, our results cannot establish causality.

Although we observed a shorter length of stay among patients mobilized on POD-0, this audit did not specifically examine the effect of early mobilization on length of stay, as it was not among the objectives of this study and would require a different research design and statistical analysis to explore that relationship. Additionally, we did not assess the impact of POD-0 mobility rates on patient outcomes such as morbidity or mortality, though this is an area we aim to investigate in the future to determine if the results further support current ERAS guidelines on mobilization.

Despite these limitations, this audit is a strong example of a three-cycle process where targeted interventions significantly improved early mobilization rates. It demonstrated the effectiveness of a multidisciplinary approach in driving improvements and adapting to guideline standards over time, ultimately contributing to enhanced postoperative care and better patient outcomes.

## Conclusions

This audit highlights the successful implementation of early mobilization standards for thoracic surgery patients at QEHB, showing the need for targeted efforts in improving ERAS adherence. Initially, only 30% of patients were mobilized on POD-0, but the rate increased to 67.6% with the implementation of MDT training and posters as visual reminders. The findings highlight the necessity of a collaborative effort among healthcare personnel to promote early mobilization, which is critical for lowering postoperative complications and improving recovery.

Factors influencing mobilization rates, such as patient arrival time from recovery and postoperative location, were identified, indicating the need for tailored interventions that address the unique characteristics of different surgical procedures. While this audit observed a correlation between early mobilization and shorter lengths of stay, further research is required to explicitly establish the causal relationship between mobilization and improved outcomes. Overall, this audit illustrates the effectiveness of targeted interventions on early mobilization and highlights the importance of a multidisciplinary approach in optimizing postoperative care by adhering to the ERAS guidelines. Furthermore, it highlights potential areas for additional interventions to improve and sustain optimal early mobilization practices, ultimately aimed at enhancing patient outcomes in thoracic surgery.
